# Drainless thoracoscopic surgery should be avoided in primary spontaneous pneumothorax with pleural adhesion

**DOI:** 10.1093/icvts/ivac237

**Published:** 2022-09-06

**Authors:** Chieh-Kuo Lin, Ka-I Leong, Cheng-Hung How, Hu-Lin Christina Wang, Chao-Yu Liu

**Affiliations:** Division of Thoracic Surgery, Department of Surgery, Far-Eastern Memorial Hospital, New Taipei City, Taiwan; Division of Thoracic Surgery, Department of Surgery, Far-Eastern Memorial Hospital, New Taipei City, Taiwan; Division of Thoracic Surgery, Department of Surgery, Far-Eastern Memorial Hospital, New Taipei City, Taiwan; Division of Thoracic Surgery, Department of Surgery, Far-Eastern Memorial Hospital, New Taipei City, Taiwan; Division of Thoracic Surgery, Department of Surgery, Far-Eastern Memorial Hospital, New Taipei City, Taiwan; Faculty of Medicine, School of Medicine, National Yang Ming Chiao Tung University, Taipei, Taiwan

**Keywords:** Chest drain, Drainless, Thoracoscopic surgery, Spontaneous pneumothorax

## Abstract

**OBJECTIVES:**

Drainless thoracoscopic surgery, defined by omitting chest drain after surgery, has been demonstrated to be feasible in selected patients for pulmonary resection. However, drainless procedure for the treatment of primary spontaneous pneumothorax has raised concerns for its safety and thus has been less often reported. We aimed to share our preliminary experience regarding how to select patients with spontaneous pneumothorax for this procedure.

**METHODS:**

A retrospective study recruiting 303 consecutive patients with the diagnosis of spontaneous pneumothorax undergoing thoracoscopic surgery in our centre from August 2016 to June 2020 was done. After careful selection, the chest drain was omitted in selected patients who underwent non-intubated uniportal thoracoscopic surgery. Patients’ clinical characteristics and perioperative outcomes were analysed.

**RESULTS:**

A total of 34 patients underwent drainless thoracoscopic surgery for the treatment of spontaneous pneumothorax. Pleural adhesion was noted in 9 patients during surgery, and all of them (100%) developed residual pneumothorax, among which intercostal drainage was required in 2 (22.2%) patients and ipsilateral pneumothorax recurred 3 years after surgery in 1 (11.1%) patient. Among the remaining 25 without pleural adhesion, 17 (68.0%) developed minor residual pneumothorax (*P *=* *0.006), which all resolved spontaneously within 1–2 weeks, with no complications or recurrence during postoperative follow-up for at least 2 years.

**CONCLUSIONS:**

Drainless thoracoscopic surgery for the treatment of primary spontaneous pneumothorax is feasible but can be risky without careful patient selection. In our experience, the drainless procedure should be avoided in patients with identifiable pleural adhesion noted during surgery.

**Figure:**
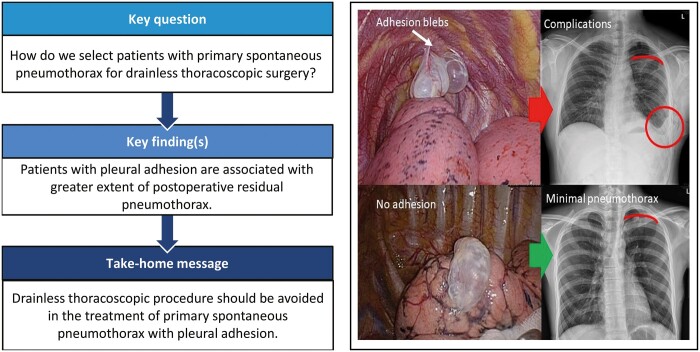

## INTRODUCTION

In recent decades, the development of video-assisted thoracic surgery (VATS) progressed consistently, including efforts to improve patient outcomes and expedite patient recovery [1]. Chest drainage catheters are routinely placed after thoracic surgery but the chest drain itself is usually the culprit for causing postoperative pain [2], limited ventilatory function [3] and interference with ambulation [4]. Previous studies have reported a ‘no-drain policy’, that is, avoiding chest drain placement after VATS [5–9] and demonstrated the feasibility and safety of this policy [10–13]. Without postoperative chest draining, complications such as pleural effusion, subcutaneous emphysema or residual pneumothorax require careful monitoring after surgery. According to the results of the previous study, postoperative residual pneumothorax may be as high as 40% [13]. Therefore, the safety of omitting the chest drain after VATS still warrants concern.

Drainless VATS combining non-intubated anaesthesia, known as ‘tubeless VATS’, has also been demonstrated to be safe in selected cases [10–14]. In our previous study investigating the safety of tubeless VATS for pulmonary wedge resection, primary spontaneous pneumothorax (PSP) was found to be a risk factor for postoperative complications such as residual pneumothorax, pleural effusion or subcutaneous emphysema [14]. However, the cause of complications and the related clinical implications remain undetermined. The safety and benefits of drainless VATS for the treatment of PSP have been demonstrated in a few literatures. However, their postoperative complications and recurrence have not yet been discussed [15, 16]. The study was to further investigate our previous findings and to analyse the complications and long-term outcome of drainless VATS for the treatment of PSP.

## MATERIALS AND METHODS

### Patient selection

This retrospective study reviewed the data of patients diagnosed with PSP who underwent VATS in our institution from September 2016 to June 2020. Patients’ medical history, preoperative routine blood examinations, electrocardiogram and chest X-ray (CXR) films were obtained before surgery.

**Figure 1:**
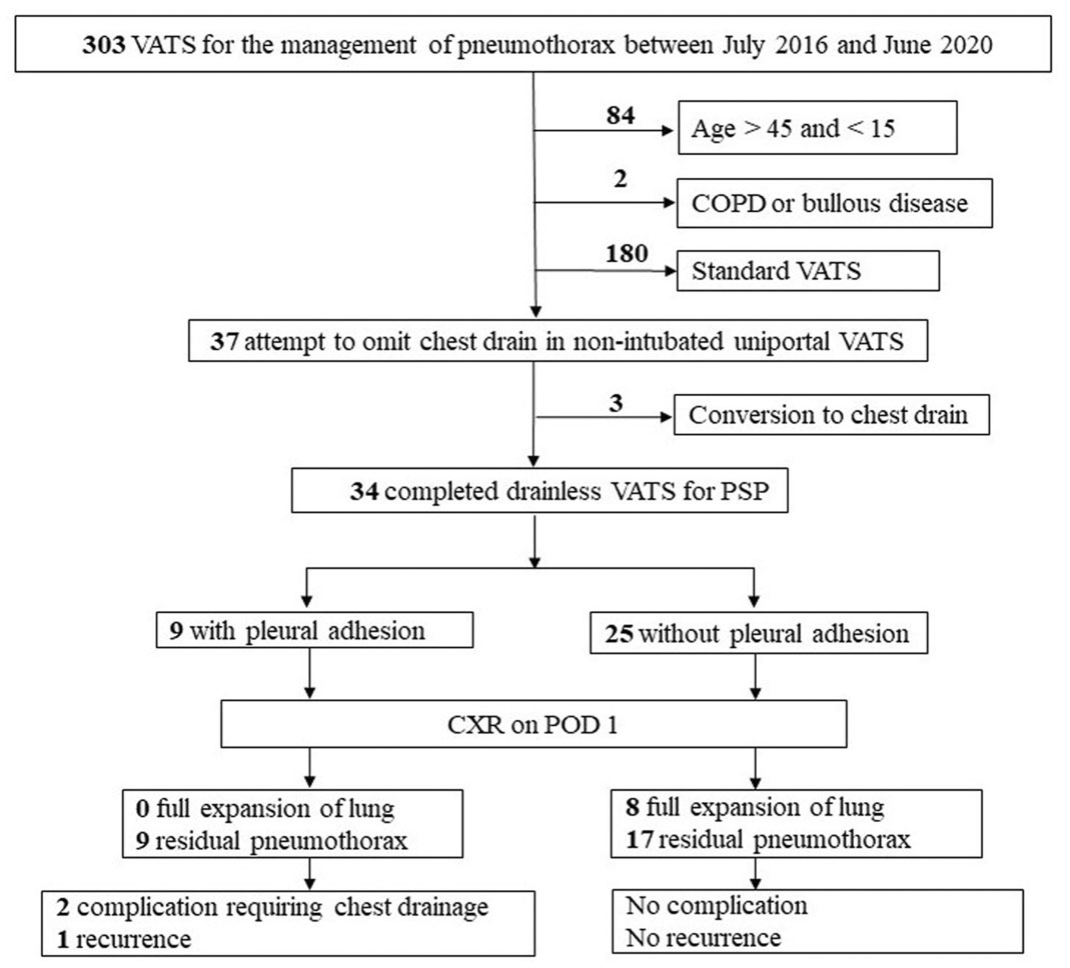
Flow chart of patient selection. COPD: chronic obstructive pulmonary disease; POD: postoperative day; PSP: primary spontaneous pneumothorax; VATS: video-assisted thoracic surgery.

Patients selected for non-intubated uniportal (NIU) VATS met the inclusion criteria, as follows: aged from 15 to 45 years, initially diagnosed or recurrent ipsilateral PSPs, without concomitant or previous malignant or infectious pleural diseases and without a history of thoracic trauma, chest surgery or severe chest wall deformity. The exclusion criteria were: previous diagnosis of chronic obstructive pulmonary disease or bullous disease, patients who were judged inappropriate for NIU VATS by anaesthesiologist or operating surgeon would undergo standard VATS procedure with multi-portal design, intubated general anaesthesia (Fig. 1). The decision to omit the placement of postoperative chest drain was mainly depended on operating surgeon’s discretion. Generally, postoperative chest drain would be placed when patients required ≥4 individual unilateral wedge resections to avoid significant residual pleural space after surgery. We would also place chest drain in patients with concomitant haemothorax >200 ml noted intraoperatively.

### Ethical considerations

The protocol for this study was approved by the institutional review board of Far East Memorial Hospital (institutional review board approval number: 109137-E, 5 October 2020). A waiver of informed consent was approved due to the retrospective nature of the study.

### Non-intubated anaesthesia and perioperative management

Patients suitable for non-intubated anaesthesia were pre-medicated with intravenous midazolam (2 mg) and fentanyl (50–100 μg). After placing each patient in the lateral decubitus position, propofol was administered via a target-controlled infusion pump (Alaris PK Syringe Pump, CareFusion, San Diego, USA) with an initial target concentration of 3.5 μg/ml. Standard perioperative monitoring such as blood pressure, electrocardiogram and pulse oximetry was performed, and function was monitored with a bispectral index sensor (Bispectral index Brain Function Monitoring, Medtronic, Dublin, Ireland) to monitor the level of consciousness as a guide to propofol dosage. During the procedure, the patients breathed spontaneously with the placement of a high-flow nasal cannula (Optiflow, Thrive, Fisher & Paykel Healthcare, Auckland, New Zealand). Prior to pulmonary resection, regional anaesthesia with intercostal nerve block was administered by a surgeon using 10 ml 0.5% bupivacaine in the intercostal space (ICS) where the incision was made, as well as 1 ICS, above and below.

### Uniportal video-assisted thoracic surgery techniques

Patients were put lateral decubitus position. After making a one 3-cm skin incision at the fourth or fifth ICS of the anterior axillary line, a wound protector (LapShield™, Lagis^®^, Taiwan) was fixed without rib spreading. Lung collapse was obtained by creating an iatrogenic pneumothorax with gentle compression by endoscopic sponges while the patient breathed spontaneously. All procedures were performed under thoracoscopic assistance, in which a 5-mm 30° thoracoscopic video camera, grasping instruments and mechanical endo-staplers were simultaneously fitted into the single incision. Pulmonary wedge resection of blebs was performed as needed using the endoscopic linear stapler device.

### Operative findings and pneumolysis

**Figure 2:**
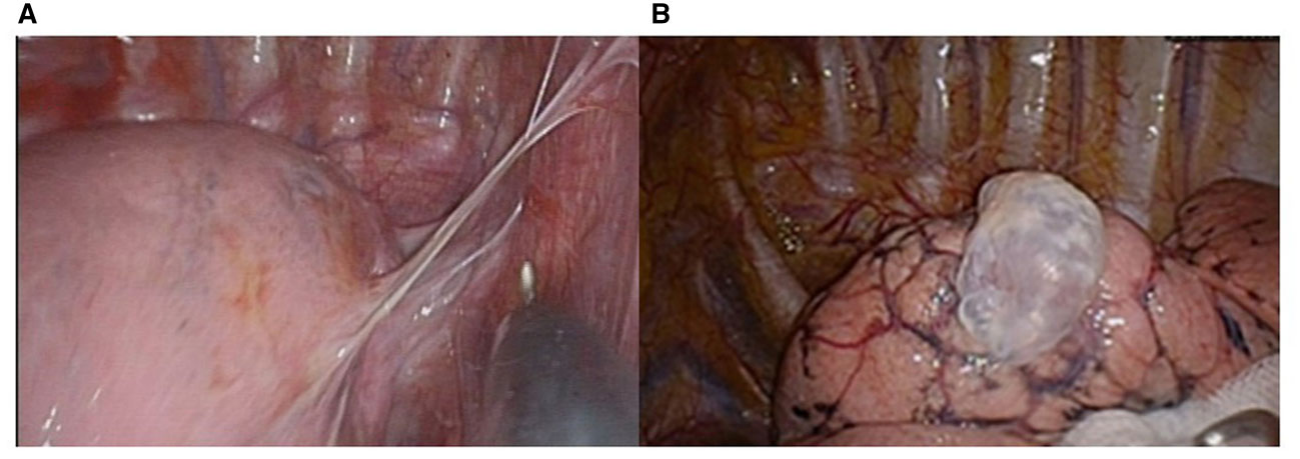
Under thoracoscopic vision, 9 patients were noted to have pleural adhesion, with adhesive band between visceral and parietal pleura, mostly found at apex of lung (**A**). Other patients had no pleural adhesion (**B**).

Under thoracoscopic vision, blebs can usually be seen at the apical pleura of the lung, with or without adhesion band formation (Fig. 2A and B). The adhesion bands needed to be cauterized and divided with monopolar cautery before performing bleb resection. Clips would be needed whenever hemostasis was required. Extrapleural dissection was rarely needed in our cohort.

### Methods of pleurodesis

Abrasion pleurodesis was performed, which involved handling with a 2 × 2 inches gauze sponge clamped by the ring forceps and scratching the parietal pleura surface above the fifth rib until a wide mild oozing and abraded pleura were seen. For patients who were devoid of abrasion pleurodesis, an absorbable polyglycolic acid (PGA) sheet (10 cm × 10 cm; Gunze Co., Kyoto, Japan) or a Vicryl mesh (8.5 cm × 10.5 cm; Ethicon, Somerville, NJ, USA) was used to cover the staple line after bullectomy at the end of surgery.

### Air-leak test manoeuvre

The air-leak test manoeuvre was described in our previous work [17]. At the end of the operation, one 12-Fr pigtail catheter was placed through the 1st ICS above the incision site into the pleural cavity. The catheter was connected to a digital drainage system (DDS) (Thopaz, Medela Healthcare, Switzerland) in which the suction pressure was maintained at the level of −20 cmH_2_O. After the incision was covered with wet gauze, the lung was re-expanded due to negative intrapleural pressure provided by DDS suction. The re-expansion process was assured under thoracoscopic vision. The single incision was then closed in layers. If the DDS revealed an air flow rate of 0 ml/min while completing the wound closure, the pigtail catheter was removed immediately. Otherwise, the pigtail catheter was left in place if any air flow was detected by DDS, and the status was defined as an intraoperative conversion to intercostal drainage (ICD). For patients in whom DDS was not used, an underwater sealing test was performed. The section of the lung with the staple line was immersed in saline poured into the pleural cavity. With manual mask ventilation performed by an anesthesiologist, the lung was expanded under thoracoscopic vision. When closing the incision, the pigtail catheter was connected to a draining tube that was immersed in water to check air leakage. If an air leak was noted, conversion to retained chest drain was conducted. Otherwise, the pigtail catheter was removed after the wound closure.

### Postoperative chest X-ray and patient follow-up

**Figure 3:**
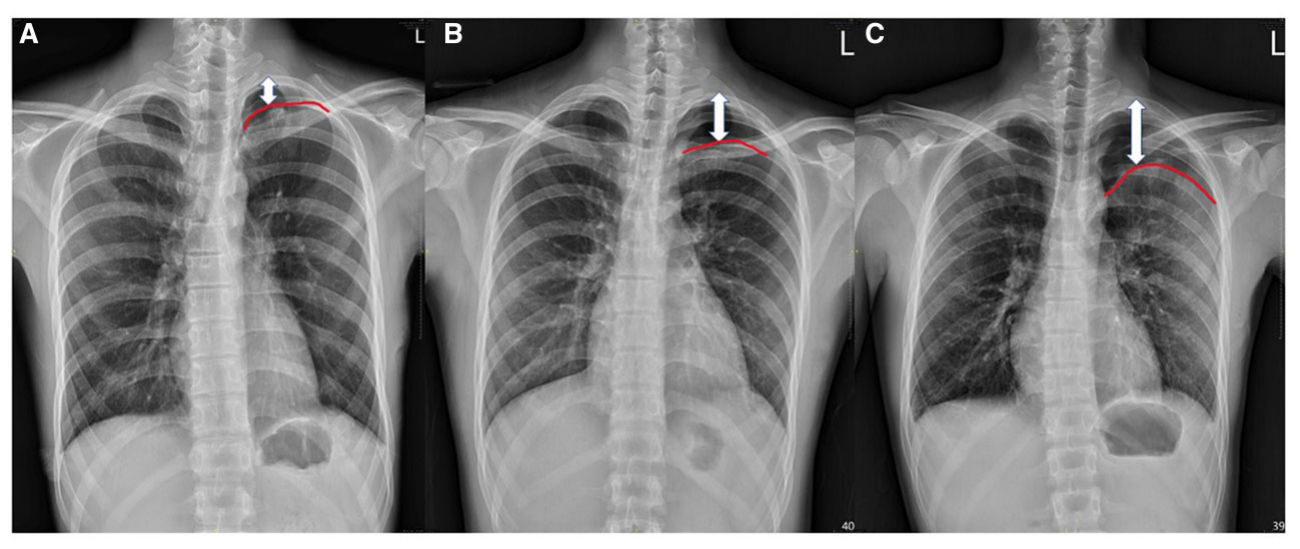
To standardize the degree of postoperative residual pneumothorax, the distance from the apical pleura (curved line) to the apex of the chest wall (double-ended arrow) was measured in the intercostal spaces (ICSs), with 1 ICS as minimal (**A**), 2 ICSs as minor (**B**) and 3 ICSs (**C**) as moderate residual pneumothorax.

To ensure lung expansion, CXR was obtained on the day of operation and on postoperative day (POD) 1. Patients were discharged home after wound pain receded and no ICD was indicated. The CXR films were interpreted by the surgeon and reviewed by the principal investigator. Residual pneumothorax was defined as an identifiable apical pleural shadow on the CXR films after surgery. To standardize the degree of residual pneumothorax, the distance from the apical pleura to the apex of the chest wall was measured in the ICSs, with 1 ICS as minimal, 2 ICSs as minor and 3 ICSs as moderate residual pneumothorax (Fig. 3A–C). All patients were closely followed for at least 2 years postoperatively.

### Statistics

The clinical characteristics of the patients and perioperative data were retrospectively collected. Continuous variables such as age are expressed as means with standard deviations. Categorical variables such as sex are presented as numbers and frequencies (%). To compare the difference between subgroups with small sample size, the Mann–Whitney *U*-test was used for continuous data and Fisher’s exact test was used for categorical data. SPSS (22.0; Inc., Chicago, IL, USA) was employed to analyse the data. All statistical tests were two-sided with 95% confidence intervals (defined by *P *<* *0·05).

## RESULTS

A total of 303 patients underwent VATS for pneumothorax from September 2016 to June 2020. Of these, 37 patients met the criteria for NIU VATS procedure. Attempts to omit the chest drain were applied to these patients, and 3 patients were converted to chest drain placement at the end of the operative procedure. A total of 34 drainless VATS were completed for the treatment of PSP (Fig. 1).

Patients’ clinical characteristics and perioperative outcomes are presented in Tables 1 and 2. Among our cohort (*n* = 34), 30 patients (88.2%) were men. The process of non-intubated anaesthesia was performed smoothly in all patients and there were no conversions to intubated general anaesthesia. Surgeries for all patients were performed smoothly using the uniportal approach. During surgery, 9 patients were found to have adhesion between visceral and parietal pleura (Fig. 2A). For those with pleural adhesion, pneumolysis was performed before resection of blebs. Blebs were mainly located at the upper lobes (Fig. 2B), with only 5 cases having left lower lobe blebs resected simultaneously. In this highly selective cohort, wedge resection numbers were limited to ≤3. Among these patients, 24 (70.6%) patients underwent only 1 wedge resection. Staple cartridges were efficiently used with a median of 2.7 ± 1.0 in the whole cohort. To avoid postoperative haemothorax, abrasion pleurodesis was applied in only 3 (8.8%) earlier cases from this cohort. For the substitution of abrasion pleurodesis at the end of the surgery, absorbable PGA sheets were used in 18 cases (52.9%) and vicryl mesh was applied in 12 cases (35.3%). The mean postoperative hospital stay was 1.9 ± 0.8 days. A total of 26 patients (76.5%) had CXR films showing residual pneumothorax on the POD1, with 12 (35.3%) minimal, 11 (32.4%) minor and 3 (8.8%) moderate pneumothorax cases based on our definition stated above.

**Table 1:** Demographic and clinical characteristics of 34 patients undergoing drainless VATS for PSP

Characteristics	*N* (%)
Age (years), mean ± SD	22.9 ± 6.9
Male	30 (88.2)
BMI, mean ± SD	19.2 ± 2.6
Non-smoker	27 (79.4)
First episode of PSP	20 (58.8)
Laterality	
Right	15 (44.1)
Left	19 (55.9)
Bleb location	
RUL	15 (44.1)
LUL	14 (41.2)
LUL + LLL	5 (14.7)
Presence of pleural adhesion	9 (26.5)

BMI: body mass index; LLL: left lower lobe; LUL: left upper lobe; PSP: primary spontaneous pneumothorax; RUL: right upper lobe; SD: standard deviation; VATS: video-assisted thoracic surgery.

**Table 2:** Perioperative data of patients with PSP with or without pleural adhesion undergoing drainless VATS

Variables	25 non-adhesion, *N* (%)	9 adhesion, *N* (%)	*P*-Value
Age (years), mean ± SD	22.96 ± 7.40	22.56 ± 5.59	0.788
BMI (kg/m^2^), mean ± SD	19.02 ± 2.10	19.61 ± 3.71	0.969
Never smoked	20 (80.0)	7 (77.8)	0.614
First episode of PSP	17 (68.0)	3 (33.3)	0.079
Number of wedge resections			
1	18 (72.0)	6 (66.7)	0.536
2	5 (20.0)	3 (33.3)	
3	2 (8.0)	0 (0)	
Number of staple cartridges used, mean (median) ± SD	2.60 (2) ± 1.04	2.89 (3) ± 0.928	0.442
Operation time (min), mean ± SD	52.60 ± 18.03	46.67 ± 12.25	0.465
Postoperative hospital stays (days), mean (median) ± SD	1.96 (2) ± 0.84	1.78 (2) ± 0.67	0.673
Method of pleurodesis			0.150
Abrasion	2 (8)	1 (11.1)	
Vicryl mesh	11 (44)	1 (11.1)	
PGA sheet	12 (48)	6 (66.7)	
None	0 (0)	1 (11.1)	
Pneumothorax on POD1 CXR			0.006
None	8 (32.0)	0 (0)	
Minimal^a^	10 (40.0)	2 (22.2)	
Minor^b^	7 (28.0)	4 (44.4)	
Moderate^c^	0 (0)	3 (8.8)	
Complication requiring ICD	0 (0)	2 (22.2)	0.064
Ipsilateral recurrence	0 (0)	1 (11.1)	0.265

a Pneumothorax with 1 intercostal space between the apical visceral pleura to the apex of chest wall.

b Two intercostal spaces between the apical visceral pleura to the apex of chest wall.

c Three intercostal spaces between the apical visceral pleura to the apex of chest wall.

BMI: body mass index; CXR: chest roentgenogram; ICD: intercostal drainage; PGA: polyglycolic acid; POD1: postoperative day 1; PSP: primary spontaneous pneumothorax; SD: standard deviation; VATS: video-assisted thoracic surgery.

Patients were separated into groups with and without pleural adhesion (Table 2). Among 9 patients with pleura adhesion, all had POD1 residual pneumothorax detected with CXRs, with 7 (53.2%) minor-to-moderate pneumothorax cases based on our definition stated above. Two of these patients required thoracocentesis because of the development of subsequent pleural effusion. One patient experienced ipsilateral pneumothorax recurrence 3 years later. However, the other 25 without pleura adhesion had less residual pneumothorax, including 8 (32.0%) with full expansion of lungs after surgery (*P *=* *0.006). Neither complication requiring ICD nor pneumothorax recurrence was reported within a minimum follow-up time of 24 months.

## DISCUSSION

In our previous study [14], we shared our initial experience with tubeless VATS for pulmonary wedge resection, concluding that the diagnosis of PSP was a risk factor for abnormal CXRs taken postoperatively. To the best of our knowledge, that study pioneered tubeless VATS for the treatment of PSP. Reviewing the literatures, omitting chest tube after standard VATS (with double lumen intubation and multi-ports access) for the treatment of PSP has been proved to be a feasible approach with the benefit of reducing postoperative pain and hospital stay [15, 16]. However, there have been no data regarding postoperative complications and their recurrence. In the present study, we further investigated the images and clinical complications, as well as long-term outcomes of those patients who had received tubeless VATS for the treatment of PSP in our institution. We found that omitting chest drain after NIU VATS is feasible for treating PSP but may still involve a level of risk without careful patient selection. It is our experience that chest drain should be placed in patients with pleural adhesion noted intraoperatively.

Omitting the chest drain after thoracic surgery has been demonstrated to be feasible in the treatment of various pulmonary diseases [5–14]. However, the technique has been less recommended in the treatment of PSP, which, by nature, is a clinical abnormality for which a chest drain is routinely used as a primary treatment. The most frequent clinical concern after drainless VATS is postoperative residual pneumothorax. The risk of further re-insertion of the chest drainage tube is typically considered [5, 7, 18, 19]. However, the residual pneumothorax may not have clinically significant complications. The presence of residual pneumothorax has an incidence of 7.6–59% in patients without postoperative chest drains and the condition requiring ICD accounted for 2.3% [17, 20, 21]. Yang *et al.* [13] reported rates of 40% and 6.6% postoperative residual pneumothorax at POD1 and at 2 weeks after VATS without chest drain, respectively. We reported residual pneumothorax after VATS without chest drain with a rate of 19.4% in another of our previous studies [17] and none of those patients required postoperative ICD. In the present study, 26 of 34 (76.5%) patients had postoperative residual pneumothorax. However, most patients (32 patients, 94.1%) resolved spontaneously by the time of OPD follow-up 1–2 weeks later.

The presence of pleura adhesion was identified as a significant risk factor for prolonged air leak [22, 23], which would potentially occur after parenchymal injury during adhesiolysis. Postoperative drainage amount and duration of chest tube stay were also significantly different in a retrospective study [24]. Pleural inflammation may occur after PSP, which leads to pleura thickening [25] and affects lung compliance. Full expansion of the lungs after drainless VATS for PSP may take more time, which could explain a greater residual air space retained after VATS without a chest drain in those with pleural adhesion in the present study. To minimize the risk of complications, the presence of intraoperative pleural adhesion needs to be a concern when omitting the chest drain after VATS for PSP.

Abrasion pleurodesis has been considered a standard surgical procedure after bullectomy to reduce the recurrence rate [26]. However, postoperative severe chest pain is a major concern and haemothorax may occur if the procedure is performed too aggressively. In the context of no drain policy, the postoperative pleural effusion or haemothorax is inevitable after abrasion pleurodesis. In the present study, we performed abrasion pleurodesis in only 3 earlier cases and noticed a development of minimal pleural effusion after surgery. Therefore, the ‘mesh pleurodesis’ with the use of Vicryl mesh or PGA mesh was adopted in the subsequent surgeries performed for patients undergoing the drainless procedure. The effects of mesh covering the pleura to reduce the PSP recurrence rate have been reported. Lee *et al.* [27] conducted a prospective, randomized, multicentre case-controlled study to compare the effects of coverage using absorbable cellulose mesh sprayed with fibrin glue with that of mechanical abrasion in patients who underwent thoracoscopic bullectomy for PSP. Results of that study revealed that the effects of staple line coverage without mechanical pleurodesis were the same as those of mechanical pleurodesis in preventing postoperative recurrence after surgical intervention for PSP. In addition, the coverage group had better recovery from pain. A rising trend of combining the use of PGA sheet and fibrin glue without abrasion pleurodesis had been published, with good postoperative outcome and low ipsilateral pneumothorax recurrence rate [28, 29]. The use of vicryl mesh has also been widely applied to prevent postoperative air-leak and reduce the recurrence rate after VATS bullectomy. Recently, 1 single-blind, prospective, randomized controlled trial demonstrated that postoperative recurrence of pneumothorax was effectively reduced by covering the staple line with vicryl mesh [30]. We believe that the use of mesh instead of mechanical abrasion is the ideal method for pleurodesis. The patient may experience less postoperative pain and residual pleural effusion can be minimized.

###  

The limitations of this study lie in its retrospective nature. First, patients were all carefully selected for omitting the chest drain, and they were relatively young and mostly non-smokers. The results need to be carefully interpreted when being applied to other patients with PSP. More studies are warranted to verify the safety and feasibility of drainless procedures. Second, the power of statistical significance was weakened by the small cohort in this study. The type II error was high. As a pioneer study, however, patient safety was our major concern. Given the results demonstrating the greater extent of residual pneumothorax in patients with pleural adhesion, the procedures were thereafter performed cautiously for a limited number of candidates, especially patients with pleural adhesion. Third, it was difficult to quantify postoperative pneumothorax because the status of pneumothorax was determined by CXR images instead of computed tomography. CXR readings are subjective by nature; thus, interobserver bias was inevitable. Fourth, using residual pneumothorax as a surrogate for complications in our study may be reasonable but lacks statistical evidence. Analysis of retrospective data limits inferences of causality; therefore, more data are needed to confirm the causal relationship between the extent of residual pneumothorax and the rate of complications. Last, the study number was too small to determine conclusively that the recurrence rate is higher in patients undergoing drainless procedures. Further prospective, comparative study with a large cohort is required to confirm the present findings.

## CONCLUSION

In conclusion, drainless VATS for the treatment of PSP is feasible but should be performed cautiously on selected patients. The presence of pleura adhesion carries higher risk of a greater extent of residual pneumothorax, which may result in more complications and recurrence. The procedure is not recommended for PSP patients with the presence of pleural adhesion.

**Conflict of interest:** none declared.

**Data availability**

The data underlying this article will be shared on reasonable request to the corresponding author.

**Author contributions**

**Chieh-Kuo Lin:** Conceptualization; Data curation; Formal analysis; Project administration; Writing—original draft; Writing—review & editing. **Ka-I Leong:** Conceptualization; Project administration; Writing—review & editing. **Cheng-Hung How:** Conceptualization; Formal analysis; Writing—review & editing. **Hu-Lin Christina Wang:** Conceptualization; Formal analysis; Writing—review & editing. **Chao-Yu Liu:** Conceptualization; Formal analysis; Writing—original draft; Writing—review & editing.

**Reviewer information**

Interactive CardioVascular and Thoracic Surgery thanks René Horsleben Petersen, Petre Vlah-Horea Botianu, Milton Saute and the other, anonymous reviewer(s) for their contribution to the peer review process of this article.

## ABBREVIATIONS

CXR: Chest X-ray
DDS: Digital drainage system
ICD: Intercostal drainage
ICSs: Intercostal spaces
NIU: Non-intubated uniportal
PGA: Polyglycolic acid
POD: Postoperative day
PSP: Primary spontaneous pneumothorax
VATS: Video-assisted thoracic surgery
